# Draft Genome Sequence of Bacillus sp. HMA207, a Strain That Exhibits β-d-Galactosidase Activity To Release Pyruvylated Galactose

**DOI:** 10.1128/MRA.01169-18

**Published:** 2018-09-13

**Authors:** Yujiro Higuchi, Hitomi Matsufuji, Kazuki Mori, Emiko Matsunaga, Kosuke Tashiro, Kaoru Takegawa

**Affiliations:** a Department of Bioscience and Biotechnology, Faculty of Agriculture, Kyushu University, Fukuoka, Japan; Georgia Institute of Technology

## Abstract

The genome sequence of the Bacillus sp. strain HMA207, the culture supernatant of which exhibited β-d-galactosidase activity to release pyruvylated galactose (PvGal), was examined to identify a PvGal-ase-encoding gene. We report here the result of whole-genome shotgun sequencing, which revealed putative PvGal-ase genes.

## Abstract (precis)

The genome sequence of the Bacillus sp. strain HMA207, the culture supernatant of which exhibited β-d-galactosidase activity to release pyruvylated galactose (PvGal), was examined to identify a PvGal-ase-encoding gene.

## ANNOUNCEMENT

In a wide range of organisms, pyruvic acid (Pv) is attached to the terminus of *N*-linked glycans (1, 2). This pyruvylation confers a negative charge and has versatile physiological roles, for example, in intercellular interactions (3). Although several enzymes involved in the biosynthesis of Pv-containing sugar chains have been reported, enzymes that metabolize Pv-attached oligosaccharides are not well understood (4–6). We previously screened soil samples and found a strain that exhibits β-d-galactosidase activity to release Pv-attached galactose (PvGal) (7). We further identified the strain to be a Bacillus species by 16S rRNA gene analysis and named the strain HMA207 (7). Here, we conducted whole-genome sequencing of Bacillus sp. strain HMA207 to identify the putative PvGal-ase genes.

The HMA207 strain was cultured in LB medium (0.5% yeast extract, 1% tryptone, 1% NaCl, and 3% agar; pH 7.0) at 30°C, with shaking at 200 rpm. For genomic DNA preparation, the ISOPLANT extraction kit (Nippon Gene, Japan) was used according to the manufacturer’s instructions. The draft genome sequence of strain HMA207 was acquired by a whole-genome shotgun-sequencing strategy using a MiSeq sequencer (Illumina, UK). With an average of 230-bp paired-end reads, a 7.78-Gbp sequence was generated from 3.38 × 10^7^ sequencing reads (1,470-fold coverage). As a quality control, we conducted trimming and filtering using Trimmomatic version 0.32 with the settings of paired-end mode (http://www.usadellab.org/cms/?page=trimmomatic). Platanus version 1.2.1 was used to assemble these sequence reads, and 18 contigs were obtained, the longest of which contained 3,953,797 bp. Genome annotation was carried out using the BLAST version 2.2.26 (blastp) nonredundant protein sequence database with an E value of <1e^−4^ and Glimmer version 3.02b with the script g3-iterated.csh. The draft genome of Bacillus sp. strain HMA207 was 5.29 Mbp, with an overall GC content of 35.1%, putatively encoding 5,416 genes with a gene density of 977 bp/gene. The mean and median gene lengths were 827 bp and 714 bp, respectively. Based on annotation of the whole-genome sequence of Bacillus sp. strain HMA207, we found three putative glycosidase genes that potentially encode PvGal-ase, named ORF1119 (GenBank accession number BEZT01000001), ORF4395 (GenBank accession number BEZT01000002), and ORF4971 (GenBank accession number BEZT01000004), located in contig01, contig02, and contig04, respectively. Further elucidation based on such gene annotation would reveal how strain HMA207 degrades PvGal-containing oligosaccharides.

### Data availability.

The contig sequences of Bacillus sp. strain HMA207 have been deposited at the DDBJ/EMBL/GenBank database under the accession numbers BEZT01000001 to BEZT01000018.

## References

[1] Spillmann D, Hård K, Thomas-Oates J, Vliegenthart JF, Misevic G, Burger MM, Finne J 1993 Characterization of a novel pyruvylated carbohydrate unit implicated in the cell aggregation of the marine sponge *Microciona prolifera*. J Biol Chem 268:13378–13387. 8514776

[2] Maruyama Y, Mikami B, Hashimoto W, Murata K 2007 A structural factor responsible for substrate recognition by *Bacillus* sp. GL1 xanthan lyase that acts specifically on pyruvated side chains of xanthan. Biochemistry 46:781–791. doi:10.1021/bi0619775. 17223699

[3] Tanaka N, Takegawa K 2001 Biosynthetic pathway and physiological role of galactose-containing oligosaccharides in fission yeast *Schizosaccharomyces pombe*. Trends Glycosci Glycotechnol 13:519–532. doi:10.4052/tigg.13.519.

[4] Yoritsune K, Matsuzawa T, Ohashi T, Takegawa K 2013 The fission yeast Pvg1p has galactose-specific pyruvyltransferase activity. FEBS Lett 587:917–921. doi:10.1016/j.febslet.2013.02.016. 23422075

[5] Yoritsune K, Higuchi Y, Matsuzawa T, Takegawa K 2014 Functional analysis of putative phosphoenolpyruvate transporters localized to the Golgi apparatus in *Schizosaccharomyces pombe*. FEMS Yeast Res 14:1101–1109. doi:10.1111/1567-1364.12207. 25195688

[6] Higuchi Y, Yoshinaga S, Yoritsune K, Tateno H, Hirabayashi J, Nakakita S, Kanekiyo M, Kakuta Y, Takegawa K 2016 A rationally engineered yeast pyruvyltransferase Pvg1p introduces sialylation-like properties in neo-human-type complex oligosaccharide. Sci Rep 6:26349. doi:10.1038/srep26349. 27194449 PMC4872226

[7] Higuchi Y, Matsufuji H, Tanuma M, Arakawa T, Mori K, Yamada C, Shofia R, Matsunaga E, Tashiro K, Fushinobu S, Takegawa K 2018 Identification and characterization of a novel β-d-galactosidase that releases pyruvated galactose. Sci Rep 8:12013. doi:10.1038/s41598-018-30508-4. 30104607 PMC6090015

